# The ChaC family of γ-glutamyl cyclotransferases is required for *Leishmania* to switch to a slow growth state and for long-term survival of the parasite

**DOI:** 10.1016/j.jbc.2022.102510

**Published:** 2022-09-17

**Authors:** Sumit Das, Puja Panja, Gaurab Chowdhury, Saroj Biswas, Yuthika Dholey, Subrata Adak

**Affiliations:** Division of Structural Biology & Bio-informatics, CSIR-Indian Institute of Chemical Biology, Kolkata, India

**Keywords:** ChaC family of γ-glutamyl cyclotransferases, GSH, trypanothione, oxidative stress, *Leishmania.*, cDNA, complementary DNA, ER, endoplasmic reticulum, FBS, fetal bovine serum, gDNA, genomic DNA, HRP, horseradish peroxidase, NAC, N-acetyl cysteine, PI, propidium iodide, ROS, reactive oxygen species, qRT-PCR, quantitative real-time PCR, sgRNA, single-guide RNA

## Abstract

The ChaC family of γ-glutamyl cyclotransferases is conserved throughout all Kingdoms and catalyzes the degradation of GSH. So far, the ChaC family proteins in trypanosomal parasites are missing in the literature. Here, we report two members of the ChaC family of γ-glutamyl cyclotransferases (LmChaC_2a_ and LmChaC_2b_) in the unicellular pathogen *Leishmania*. Activity measurements suggest that these proteins catalyze degradation of GSH but no other γ-glutamyl peptides. Recombinant LmChaC_2a_ protein shows ∼17-fold lower catalytic efficiency (*k*_cat_ ∼ 0.9 s^−1^) than LmChaC_2b_ (*k*_cat_ ∼ 15 s^−1^), although they showed comparable *K*_*m*_ values (∼1.75 mM for LmChaC_2a_ and ∼2.0 mM for LmChaC_2b_) toward GSH. qRT-PCR and Western blot analyses suggest that the LmChaC_2a_ protein was found to be constitutively expressed, whereas LmChaC_2b_ was regulated by sulfur stress. To investigate its precise physiological function in *Leishmania*, we generated overexpressed, knockout, and complement cell lines. Flow cytometric analyses show the presence of a higher intracellular GSH concentration and lower intracellular ROS level, indicative of a more reductive environment in null mutants. We found LmChaC2-expressing cells grow in GSH-containing sulfur-limited media, while the null mutants failed to grow, suggesting that LmChaC2 is crucial for cell growth with GSH as the only sulfur source. Null mutants, although reach the stationary phase rapidly, display impaired long-term survival, indicating that LmChaC2-mediated GSH degradation is necessary for prolonged survival. *In vivo* studies suggest that LmChaC2-dependent controlled GSH degradation promotes chronic infection by the parasite. Altogether, these data indicate that LmChaC2 plays an important role in GSH homeostasis in *Leishmania*.

Generally, GSH displays extreme diverse roles in prokaryotic and eukaryotic cells including mitochondrial iron-sulfur biogenesis (1), elimination of oxidative stress, metal and xenobiotic detoxification, redox buffer, storage and transport of sulfur (2, 3), and its capacity to regulate protein function by glutathionylation (4).

In addition, GSH also regulates immune cell functions, like initiating the Th1 or Th2 immune responses by controlling the antigen-processing machinery in antigen-presenting cells (5). Furthermore, the depletion of GSH is one of the markers of apoptosis (6) and low GSH concentration, are closely associated with several diseases (7). On the other hand, excessive GSH levels are also detrimental to the cell (8, 9, 10). It is well known that GSH synthesis and degradation play a significant role in maintaining GSH homeostasis (11, 12).

Protozoan parasites, trypanosomatids, have several unique features; one of them is trypanothione [T(SH)_2_] based redox metabolism. A massive amount of reactive oxygen species (ROS) is produced by activated macrophages as initial line of defense against the parasites (13, 14). Surviving parasite is able to tolerate the ROS during its life cycle by recruiting a number of antioxidant molecules including unique redox proteins and low molecular weight thiols (15). The main ROS detoxification system in trypanosomatids is a chain of reactions where tryparedoxin reduces 2-Cys-peroxiredoxin and GSH peroxidase–like enzymes sequentially after receiving reducing equivalents from trypanothione (a GSH-spermidine conjugate) (16, 17, 18). While the GSH biosynthesis steps of the parasite are common to mammals, the T(SH)_2_ biosynthesis pathways are only present in trypanosomatids (19, 20). Both γ-glutamylcysteine synthetase and GSH synthetase in the parasite’s GSH biosynthesis pathway are known (21, 22), whereas the breakdown of GSH to amino acids is completely unknown in this parasite.

ChaC (cation transport regulator-like protein) is the third gene of CHA operon in *Escherichia coli* and is supposed to function as a γ-glutamyl cyclotransferase (23). The activity of ChaC protein toward the reduced form of GSH involves the cleavage of γ-glutamyl bond of GSH with subsequent production of 5-oxoproline and Cys-Gly (24). Human ChaC1 protein has been shown to be induced under endoplasmic reticulum (ER) stress condition in the cells (25, 26). Two ChaC homologs, ChaC1 and ChaC2, are found in higher eukaryotes, where constitutively expressed ChaC2 protein displays ∼10 to 20 times reduced catalytic efficiency compared to inducibly expressed ChaC1 protein (27).

We have found two putative ChaC-like proteins of different molecular weights (237 amino acids and 323 amino acids) in tandem array within *Leishmania major* genome database. Those putative ChaC-like proteins are present in the genome database of all trypanosomal parasites. Although the eukaryotic ChaC1 and ChaC2 are the GSH-degrading enzymes (26, 27), the potential functions of leishmanial putative ChaC proteins have not yet been explored. In this article, we characterize the two ChaC proteins from *L. major* (LmChaC_2a_ and LmChaC_2b_), which catalyze the γ-glutamyl bond cleavage of the GSH to produce Cys-Gly and 5-oxoproline. Surprisingly, our data revealed that the LmChaC_2b_ (larger protein) showed ∼17 fold more catalytic activity compared to the LmChaC_2a_ (smaller protein). Although LmChaC_2b_ protein expression was significantly increased in sulfur-limited media and stationary phase culture, LmChaC_2a_ protein is constitutively expressed. In addition, we demonstrate for the first time that the growth rate of null mutants is very high but they display impaired long-term survival in the aged culture and reduced pathogenicity in macrophage and BALB/c mice.

## Results

### Sequence analysis of LmChaC_2a_ and LmChaC_2b_ proteins

**Figure 1:**
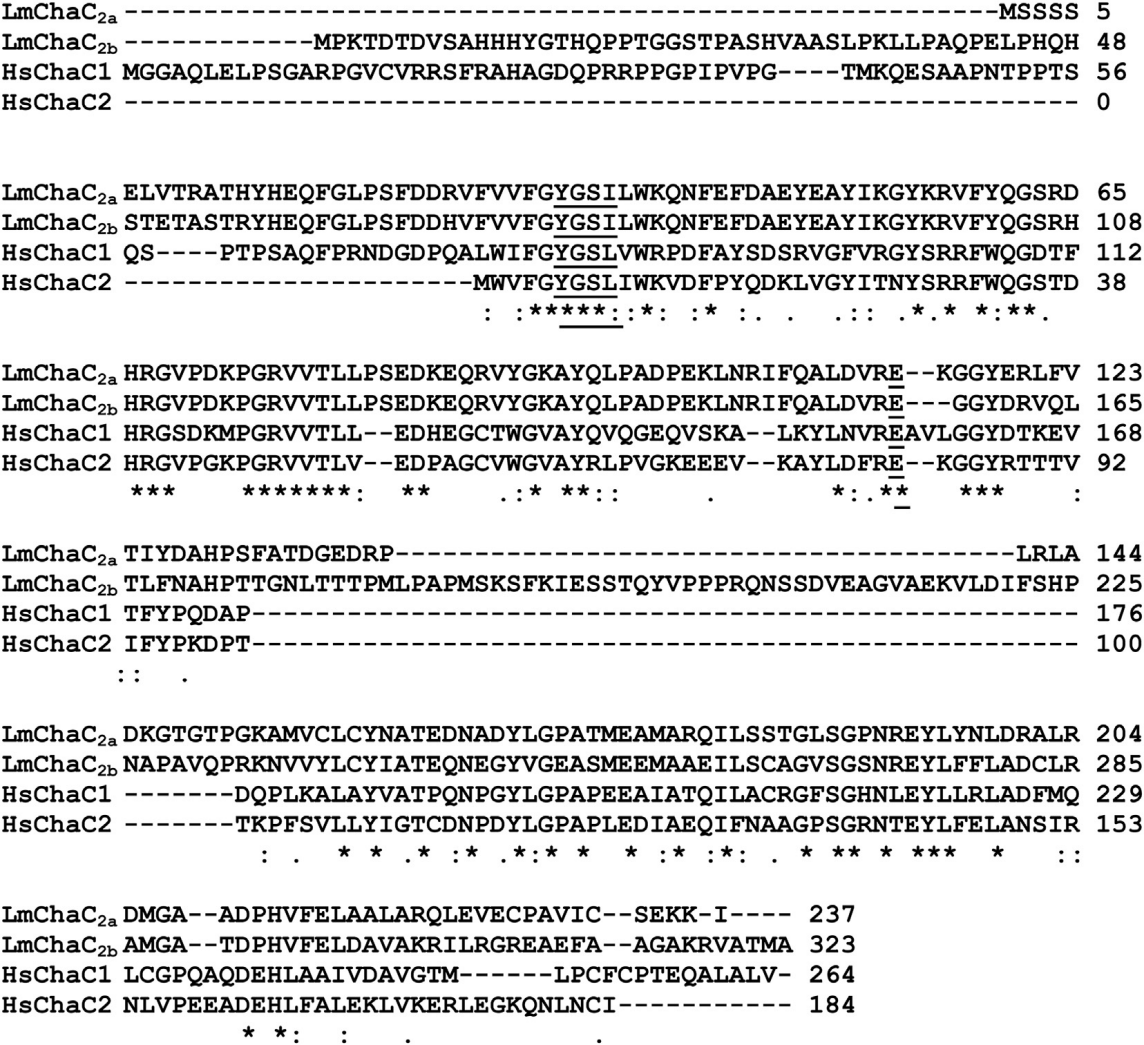
**Sequence analysis of ChaC family of γ-glutamyl cyclotransferases from *Leishmania major*.** Amino acid sequence of LmChaC_2a_ and LmChaC_2b_ were aligned with human ChaC1 and ChaC2. ∗indicates identical residue. Dashes indicate variations in sequence length among aligned proteins. Underline denotes active site residues.

Two putative LmChaC sequences in tandem array (systematic name: LmjF.22.1190 and LmjF.22.1200) within *L. major* genome database (https://tritrypdb.org/tritrypdb/app) have been identified as ORF, comprising of 237 and 323 residues, respectively. Multiple sequence alignment revealed that the shorter version (237 amino acids) and the longer version of putative LmChaC proteins (323 amino acids) bear 32% and 35% identity with the human ChaC1, respectively (Fig. 1). On the other hand, the shorter version and the longer version of putative LmChaC protein have about 40% and 45% identity with the human ChaC2, respectively (Fig. 1). Signature YGSL/I motif and catalytic glutamate residue were present in both genes, confirming that both proteins belong to the ChaC family of γ-glutamyl cyclotransferase proteins. As higher sequence identity is observed with human ChaC2 protein, we have denoted the shorter version (237 amino acids) as LmChaC_2a_ and the longer version (323 amino acids) as LmChaC_2b_.

### Biochemical characteristics of LmChaC_2a_ and LmChaC_2b_ proteins

**Figure 2:**
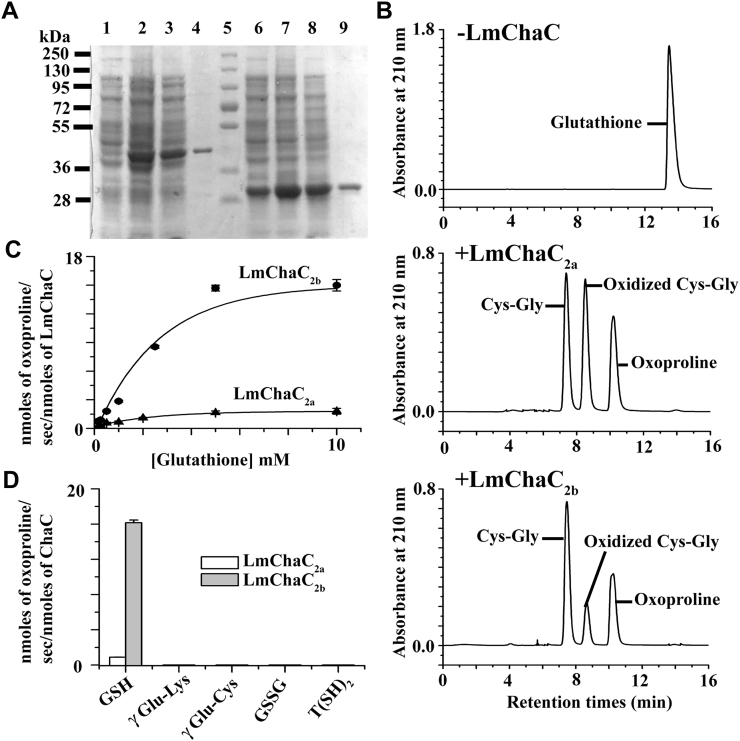
**Biochemical characteristics of LmChaC**_**2a**_**and LmChaC**_**2b**_**.***A*, SDS-PAGE. Lanes: 1, -IPTG (LmChaC_2b_); 2, +IPTG (LmChaC_2b_); 3, lysate (LmChaC_2b_); 4, purified LmChaC_2b_ protein; 5, molecular weight marker; 6, −IPTG (LmChaC_2a_); 7, +IPTG (LmChaC_2a_); 8, lysate (LmChaC_2a_); and 9, purified (LmChaC_2a_). *B*, the LmChaC proteins display enzymatic activity specifically toward GSH producing 5-oxoproline and Cys-Gly. Assay mixture was analyzed using HPLC system on a Sunfire C_18_ column (5 μm 4.6 × 250 mm, Waters) and a mobile phase of 2% (v/v) aqueous perchloric acid at 1.0 ml/min flow rate. *C*, GSH-dependent oxoproline production. All values were derived from time-dependent oxoproline production curve at different concentration of GSH in SI Fig. S1. The initial velocity of LmChaC_2a_ and LmChaC_2b_ was calculated within 5 min and 2 min incubation period, respectively. *D*, LmChaC activity in the presence of different γ-glutamyl dipeptides, GSSG and T(SH)_2_. The concentrations of T(SH)_2_, GSSG, γ-Glu- Lys, γ-Glu-Cys, LmChaC_2a_, and LmChaC_2b_ used were 10 mM, 10 mM, 10 mM, 10 mM, 3.57 μM, and 0.26 μM, respectively.

To identify the biochemical properties of LmChaC_2a_ and LmChaC_2b_ proteins, both the full length proteins were expressed in *E. coli* cells. Purified LmChaC_2b_ and LmChaC_2a_ proteins migrated to positions in the gel as predicted by theoretical relative molecular weight of 38 kDa (Fig. 2*A*, lane 4) and 28 kDa protein (Fig. 2*A*, lane 9), respectively. Products of the assay mixture were identified by HPLC after the enzymatic reaction of these LmChaC2 family proteins on GSH. When these proteins are present in assay mixture, peaks of GSH disappeared and new peaks for 5-oxoproline and Cys-Gly dipeptide emerged (Fig. 2*B*). Production of 5-oxoproline from GSH degradation confirmed the γ-glutamyl cyclotransferase activity of the LmChaC family proteins. Both LmChaC_2a_ and LmChaC_2b_ proteins follow standard Michaelis–Menten kinetics with respect to the substrate (GSH) (Figs. 2*C* and S1). The *K*_*m*_ values for GSH were very similar between LmChaC_2a_ and LmChaC_2b_ proteins: 1.75 ± 0.08 mM for LmChaC_2a_ and 2.0 ± 0.15 mM for LmChaC_2b_. These *K*_*m*_ values were comparable with human as well as mouse ChaC protein (27). Our experimental *K*_*m*_ value of recombinant LmChaC2 proteins is high with respect to intracellular concentration of GSH (0.21 mM in promastigotes and 0.39 mM in amastigotes stage (28)). One possible reason is that either the *K*_*m*_ value of natural LmChaC2 within intracellular environment might be lower compared to experimental value of recombinant protein or the previous determinations of intracellular GSH concentrations may not be sufficiently reliable. Although *K*_*m*_ of both proteins are similar but LmChaC_2a_ proteins show ∼17-fold lower catalytic efficiency (*k*_cat_ = 0.9 ± 0.1 s^−1^) than LmChaC_2b_ (*k*_cat_ = 15 ± 1.8 s^−1^). To evaluate the relative activities of the LmChaC2 proteins toward different γ-Glu-dipeptides, T(SH)_2_, and GSSG, we estimated their γ-glutamyl cyclotransferase activity by measuring the 5-oxoproline concentration (Figs. 2*D* and S2). The results showed that both enzymes showed activity toward GSH (Fig. 2*B*) but no activity toward the other γ-Glu-amino acids as well as GSSG and T(SH)_2_ (Figs. 2*D* and S2). These results suggest that both LmChaC2 proteins serve as γ-glutamyl cyclotransferases acting specifically on GSH degradation but no other γ-glutamyl peptides.

### Regulation of LmChaC_2a_ and LmChaC_2b_ proteins in *Leishmania* parasite

**Figure 3:**
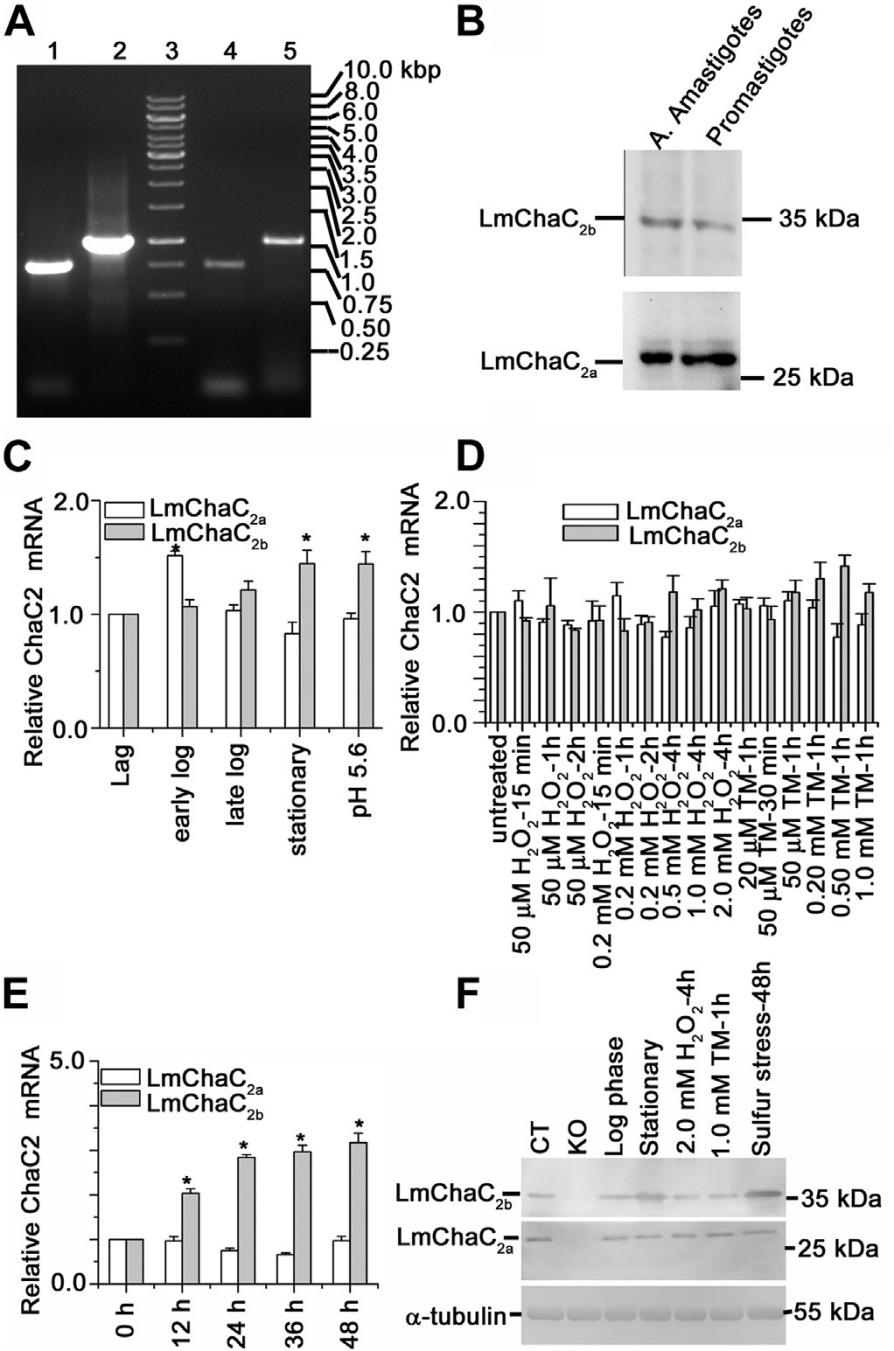
**mRNA and protein expression of LmChaC**_**2a**_**as well as LmChaC**_**2b**_**in the promastigoteand the amastigotestages of parasite.***A*, agarose gel analysis of PCR-amplified products from cDNA of LmChaC_2a_ and LmChaC_2b_. Lane 3 shows the molecular mass marker, lanes 1 and 2, correspond to PCR product from cDNA of promastigotes with LmChaC_2a_ and LmChaC_2b_ specific primers, respectively. Lanes 4 and 5, correspond to PCR product from cDNA of purified intracellular amastigotes using LmChaC_2a_ and LmChaC_2b_ specific primers, respectively. *B*, the lysate of promastigotes and the axenic amastigotes (A. Amastigotes) stages of *L. major* was used for Western blotting. Rabbit anti-LmChaC_2a_ and anti-LmChaC_2b_ antibody are used in Western blot analysis. *C*, the measurement of gene transcript abundance was analyzed by using quantitative real-time PCR (qRT-PCR) at different stages during growth of promastigotes. pH 5.6 denoted stationary phase promastigotes those were incubated at acidic pH 5.6 (pH of phagolysosome) for 24 h. *D*, the measurement of mRNA abundance in promastigotes stage of parasite was analyzed by using qRT-PCR in presence of H_2_O_2_ and ER stress inducer tunicamycin. *E*, the measurement of mRNA transcript in promastigotes stage of parasite, which incubated at sulfur-limited media up to 48 h. *F*, gene expression was analyzed by Western blot in presence of sulfur-limited media (for 48 h), H_2_O_2_ (2 mM for 4 h), or tunicamycin (1 mM for 1 h). All the data are representative of at least three independent experiments. ∗Statistically significant value of less than 0.05. cDNA, complementary DNA; ER, endoplasmic reticulum.

Earlier researchers have reported that the human ChaC1 expression was induced by ER stress or sulfur starvation, whereas human ChaC2 was constitutively expressed (25, 27). To identify whether GSH degrading enzymes, LmChaC_2a_ and LmChaC_2b_, are constitutively or inducibly expressed in the parasite, we performed semi–quantitative real-time PCR (sqRT-PCR) and Western blot analyses. The results suggested that both the LmChaC_2a_ and the LmChaC_2b_ were expressed in the promastigote as well as the amastigote stages (Fig. 3, *A* and *B*). qRT-PCR analysis data suggested that LmChaC_2b_ expression does not change significantly during the log phase of growth but increases 1.5 times during stationary phase with or without treatment with acidic pH 5.6 (Fig. 3*C*). However, treatments with neither tunicamycin (ER stress-inducing agent) nor H_2_O_2_ (oxidative stress inducer) could significantly influence the expression of either of the proteins (Fig. 3*D*). The breakdown of GSH also provides sulfur for the cells. Therefore, we used qRT-PCR and Western blot analysis to investigate the effect of sulfur-limited media on the expression of LmChaC_2a_ and LmChaC_2b_ (Fig. 3, *E* and *F*). We found that LmChaC_2b_ transcription was significantly increased (3.5-fold) in sulfur-limited media up to 48 h (Fig. 3*E*), but after 48 h, the level of LmChaC_2b_ started decreasing (data not shown), which might be caused by a decrease in cell viability. On the other hand, there was no significant change in the mRNA levels of LmChaC_2a_. We also assessed the protein expression levels by using Western blot technique where LmChaC_2b_ expression increased significantly at 48 h incubation in sulfur-limited media but LmChaC_2a_ level remained constant (Fig. 3*F*). These results indicate that LmChaC_2a_ protein was constitutively expressed, whereas LmChaC_2b_ was controlled by sulfur stress.

### Characterization of control (CT), ChaC_2a_ overexpressed (OE_2a_), ChaC_2b_ overexpressed (OE_2b_), knockout (KO), ChaC_2a_ complement (CM_2a_), ChaC_2b_ complement (CM_2b_), and ChaC_2a_ & ChaC_2b_ double complement (CM) cells

**Figure 4:**
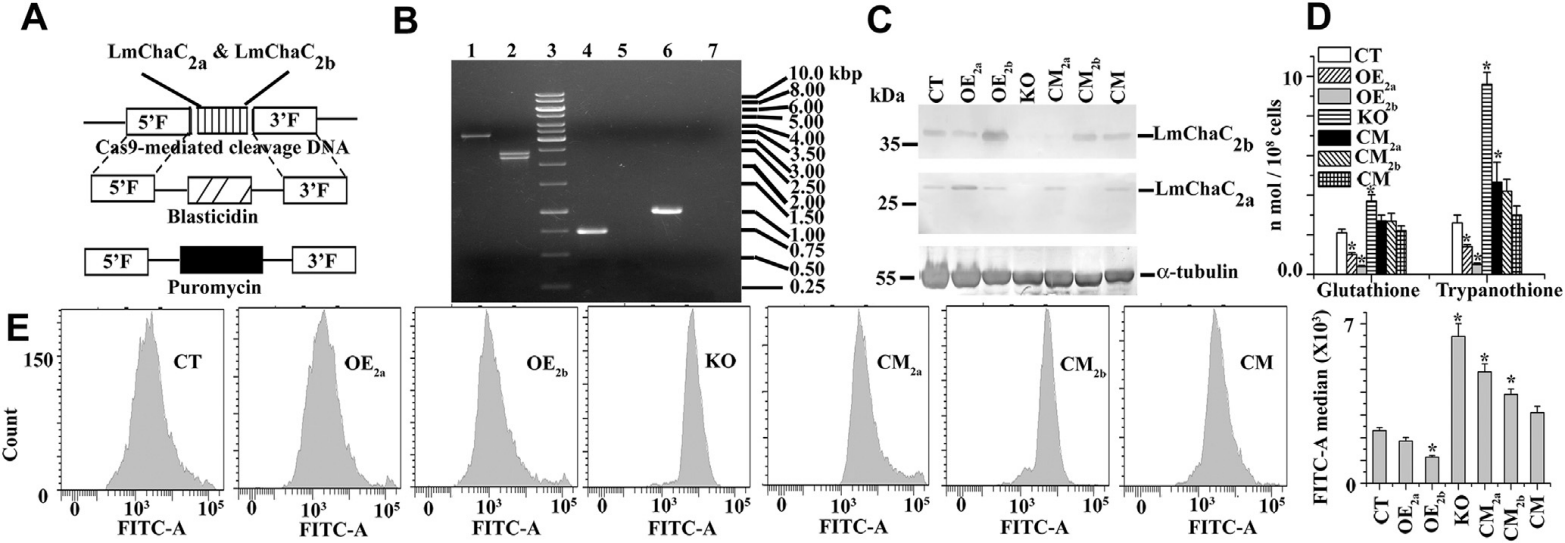
**Generation of stable KO strain for both LmChaC**_**2a**_**and LmChaC**_**2b**_**alleles by CRISPR/Cas9 based editing**. *A*, schematic representation of the LmChaC_2a_ and LmChaC_2b_ locus and the plasmid constructs used for gene replacement. *B*, agarose gel analysis of PCR amplified products of LmChaC_2a_ and LmChaC_2b_ genes. Lanes 1/4/6 and 2/5/7 correspond to PCR with gDNA from WT and KO mutants, respectively, with primers external (5′ and 3′ flanking region) (lanes 1–2) of both genes, internal of LmChaC_2a_ gene (lanes 4–5) and internal of LmChaC_2b_ gene (lanes 6–7). The expected size of the LmChaC_2a_-LmChaC_2b_ genes, PURO, and BLAST gene PCR product are 3.2, 2.4, and 2.3 Kb, respectively. The expected size of the PCR product with internal primers of LmChaC_2a_ and LmChaC_2b_ are 0.714 and 0.972 Kb, respectively. *C*, Western blot analysis was done by using anti-LmChaC_2a_, anti-LmChaC_2b_, and anti-α-tubulin antibody. 200 μg of *L. major* lysate were used for Western blotting. *D*, intracellular GSH and T(SH)_2_ measurement by HPLC system. Cell lysate was making from 2 × 10^8^ promastigotes at 4 °C and then the supernatant was filtered through 0.22 μm Millipore filter paper. Forty microliters sample was injected to the HPLC analysis using C_18_ HPLC column (Sunfire C_18_ 5 μm 4.6 × 250 mm Column, Waters) with a 2% (v/v) aqueous solution of perchloric acid used as the mobile phase with 1 ml/min flow rate at 210 nm. Peak of GSH and T(SH)_2_ were appeared at ∼12 and ∼52 min, respectively (HPLC chromatogram was shown in SI Fig. S3). GSH and T(SH)_2_ were identified with authentic standards. The intracellular concentration of GSH and T(SH)_2_ was measured from standard curve. *E*, flow cytometric determination of intracellular GSH by intracellular GSH Detection Assay Kit (Abcam). All the data are representative of at least three independent experiments. ∗Statistically significant value of less than 0.05. gDNA, genomic DNA.

To investigate the LmChaC_2a_ and LmChaC_2b_ function in *L. major*, CRISPR/Cas9 gene editing technique was used (Fig. 4*A*) to knock the genes out. PCR analysis from genomic DNA (gDNA) and Western blotting confirmed that null mutant cells (KO) no longer expressed LmChaC_2a_ or LmChaC_2b_ gene (Fig. 4, *B* and *C*). The OE_2a_ and OE_2b_ cell lines expressed higher amounts of LmChaC_2a_ and LmChaC_2b_ proteins compared to CT cell line, respectively. Western blot analysis demonstrated that the CM_2a_ cell line expressed only LmChaC_2a_ protein, whereas CM_2b_ cell line expressed only LmChaC_2b_ protein. The double complemented (CM) leishmanial cell lines expressed both the proteins in amounts comparable to control cells. Next, we investigated whether the levels of intracellular GSH and T(SH)_2_ were changed in the OE_2a_, OE_2b_, KO, CM_2a_, and CM_2b_ cells compared to CT and CM cells (Figs. 4*D* and S3). HPLC analysis suggested that KO cells had ∼2- and ∼4-fold higher intracellular GSH and T(SH)_2_ content compared to CT cells, respectively. Flow cytometry results confirmed that KO cells had higher amount of intracellular GSH (Fig. 4*E*) compared to CT, OE_2a_, OE_2b_, CM_2a_, CM_2b_, or CM cell lines. As expected, the order of intracellular GSH accumulation was KO > CM_2a_ > CM_2b_ > CT or CM > OE_2a_ > OE_2b_ cells.

**Figure 5:**
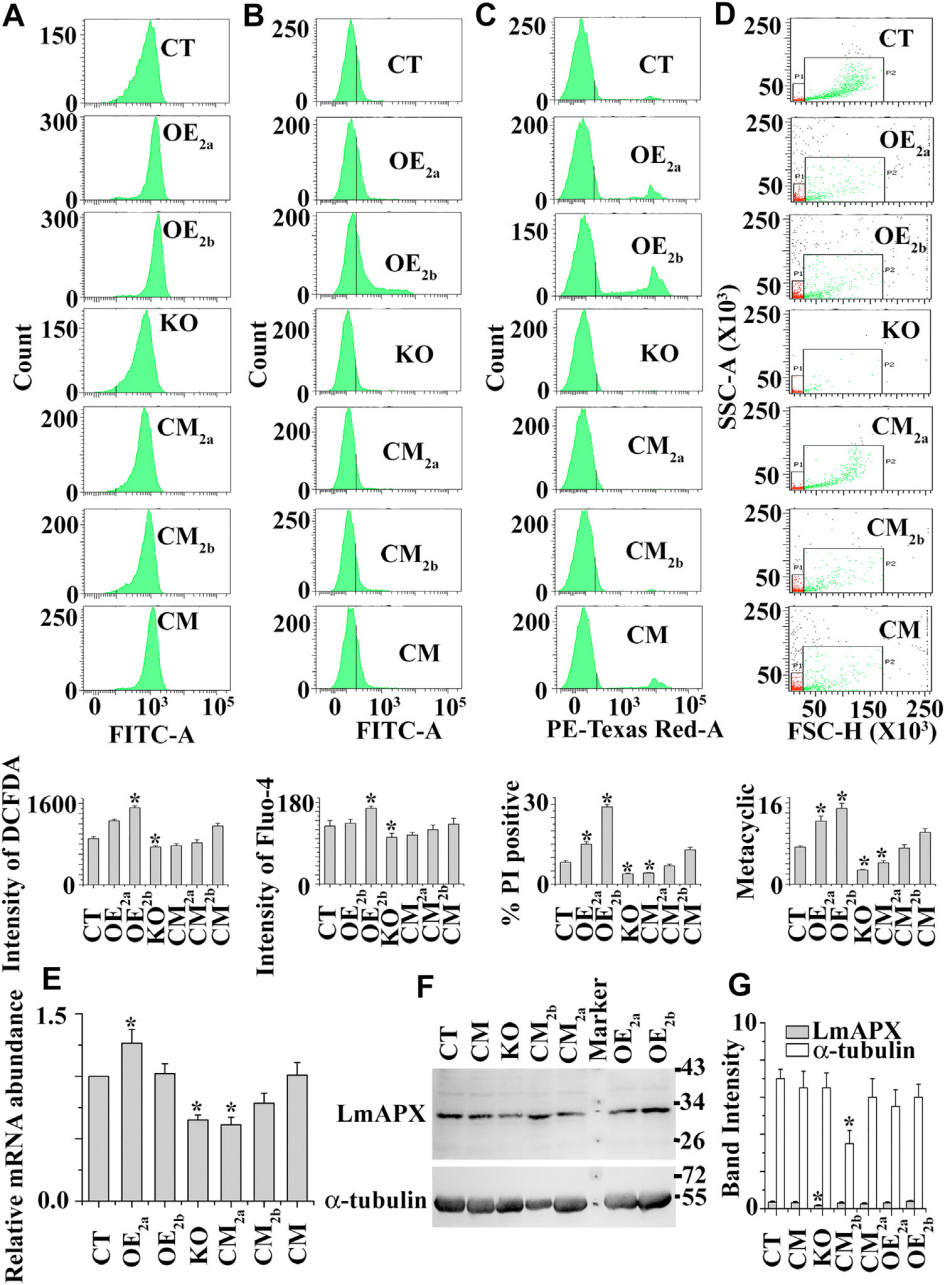
**Characteristics of control (CT), ChaC**_**2a**_**overexpressed (OE**_**2a**_**), ChaC**_**2b**_**overexpressed (OE**_**2b**_**), KO, ChaC**_**2a**_**complement (CM**_**2a**_**), ChaC**_**2b**_**complement (CM**_**2b**_**), and ChaC**_**2a**_**& ChaC**_**2b**_**complement (CM) cells.***A*, flow cytometric determination of intracellular ROS by DCFDA. *B*, the intracellular Ca^2+^ level was measured by flow cytometric method using Fluo 4AM. *C*, percentage of death cells (PI positive staining) was measured in the late log phase population by flow cytometric analysis. *D*, metacyclic (PNA negative) population in the late log phase cells were analyzed by flow cytometry after their purification using PNA. The gating on the dot-plots corresponds to different cell sizes and forward-angle light scatter (FSC) intensities (FSC_low_ and FSC_high_). FSC_low_ (denoted by small box area) represents mainly the metacyclic (PNA^−^) population. *E*, comparative studies of mRNA abundance of LmAPX among CT, OE_2a_, OE_2b_, KO, CM_2a_, CM_2b_, and CM promastigotes were analyzed by using quantitative real-time PCR. *F*, Western blot analysis was done by using anti-LmAPX and anti-α-tubulin antibody. Two hundred micrograms of *L. major* lysate were used for Western blotting. *G*, bar diagram depicted as the percentage of band intensities in panel (*F*). All the data are representative of at least three independent experiments. ∗Statistically significant value of less than 0.05. PI, propidium iodide; ROS, reactive oxygen species.

Depletion of cellular GSH and T(SH)_2_ often leads to an increased level of ROS within the cell. To evaluate the intracellular ROS concentration in CT, OE_2a_, OE_2b_, KO, CM_2a_, CM_2b_, and CM late log phase cell lines, we used DCFDA (fluorescent probe). The intracellular ROS level in the KO population was lower compared to the CT, OE_2a_, OE_2b_, CM_2a_, CM_2b_, and CM cells, indicating that the LmChaC2 proteins were able to enhance the endogenous ROS generation by degradation of GSH in the *Leishmania* parasite (Fig. 5*A*). The importance of intracellular ROS in maintaining intracellular Ca^2+^ homeostasis is now widely acknowledged. We measured intracellular Ca^2+^ levels in OE_2a_, OE_2b_, CT, KO, CM_2a_, CM_2b_, and CM cell lines by flow cytometry assay using the calcium sensor, Fluo 4AM dye (Fig. 5*B*). The intensity of the green fluorescence of Fluo 4AM dye for the KO cells was at least two folds lower than that in the CT cells, suggesting that deletion of LmChaC2 genes protected the cells against oxidative stress–mediated increase of cytosolic Ca^2+^ (Fig. 5*B*). The order of elevation of cytosolic Ca^2+^ accumulation was KO < CM_2a_ < CM_2b_ < CT or CM < OE_2a_ < OE_2b_ cells. Cell death can result from disruption of ROS homeostasis and an increase in cytosolic Ca^2+^. To investigate whether depletion of LmChaC_2a_ and LmChaC_2b_ genes shielded against oxidative stress–induced cell death, we compared the percentage of viable (propidium iodide [PI] negative) parasites among OE_2a_, OE_2b_, CT, KO, CM_2a_, CM_2b_, and CM late log phase cell lines by flow cytometry assay using the PI dye (Fig. 5*C*). Flow cytometry data revealed that the majority of the null mutant parasite population was PI negative (Fig. 5*C*), while higher amount of PI positive cells was found in the OE population, where excess amounts of LmChaC2 proteins had resulted in reduced amount of GSH. These results suggested that deletion of LmChaC2 genes protected cells against cell death at late log phase cell lines. We next tested whether deletion of LmChaC2 proteins inhibits the oxidative stress–induced cell differentiation from procyclic (noninfective stage) to nondividing metacyclic (infective stage) promastigotes. By negative agglutination reaction with peanut agglutinin (PNA), we were able to isolate metacyclic promastigotes from OE_2a_, OE_2b_, CT, KO, CM_2a_, CM_2b_, and CM late log phase cell cultures (PNA). While procyclic parasites readily agglutinate, metacyclic promastigotes, due to developmental changes in terminally exposed oligosaccharides on the lipophosphoglycan, do not interact with peanut agglutinin (PNA^-^) (29). Interestingly, a quantitative analysis of PNA agglutination by flow cytometry revealed that KO cell population consisted of lower metacyclic (PNA-) parasites compared to OE_2a_, OE_2b_, CT, CM_2a_, CM_2b_, or CM cells (Fig. 5*D*). These results strongly suggested that deletion of LmChaC2 genes prevent the cell differentiation from procyclic (noninfective stage) to metacyclic (infective stage) promastigote cells.

### Deletion of LmChaC_2a_ and LmChaC_2b_ alleles leads to decreased expression of the antioxidant gene LmAPX

The upregulation of antioxidant gene, ascorbate peroxidase (LmAPX), in the *Leishmania* parasite by oxidative stress has been reported (30). To examine the possibility that LmAPX gene expression in parasite was downregulated by reductive environment, we performed qRT-PCR and Western blot to measure LmAPX in KO, OE_2a_, OE_2b_, CT, CM_2a_, CM_2b_, and CM cells. qRT-PCR results suggested a lower expression of LmAPX (2-fold) mRNA in null mutants compared to CT or CM cells (Fig. 5*E*). The order of ascorbate peroxidase expression was KO < CM_2a_ < CM_2b_ < CT or CM < OE_2a_ < OE_2b_ cells. Western blot analysis was performed (Fig. 5, *F* and *G*) to further validate these data, and the results were remarkably similar to qRT-PCR data. Altogether, these results indicated that LmAPX was repressed under reducing environment.

### GSH homeostasis is essential for slow growth and long-term survival of *L. major* promastigotes

**Figure 6:**
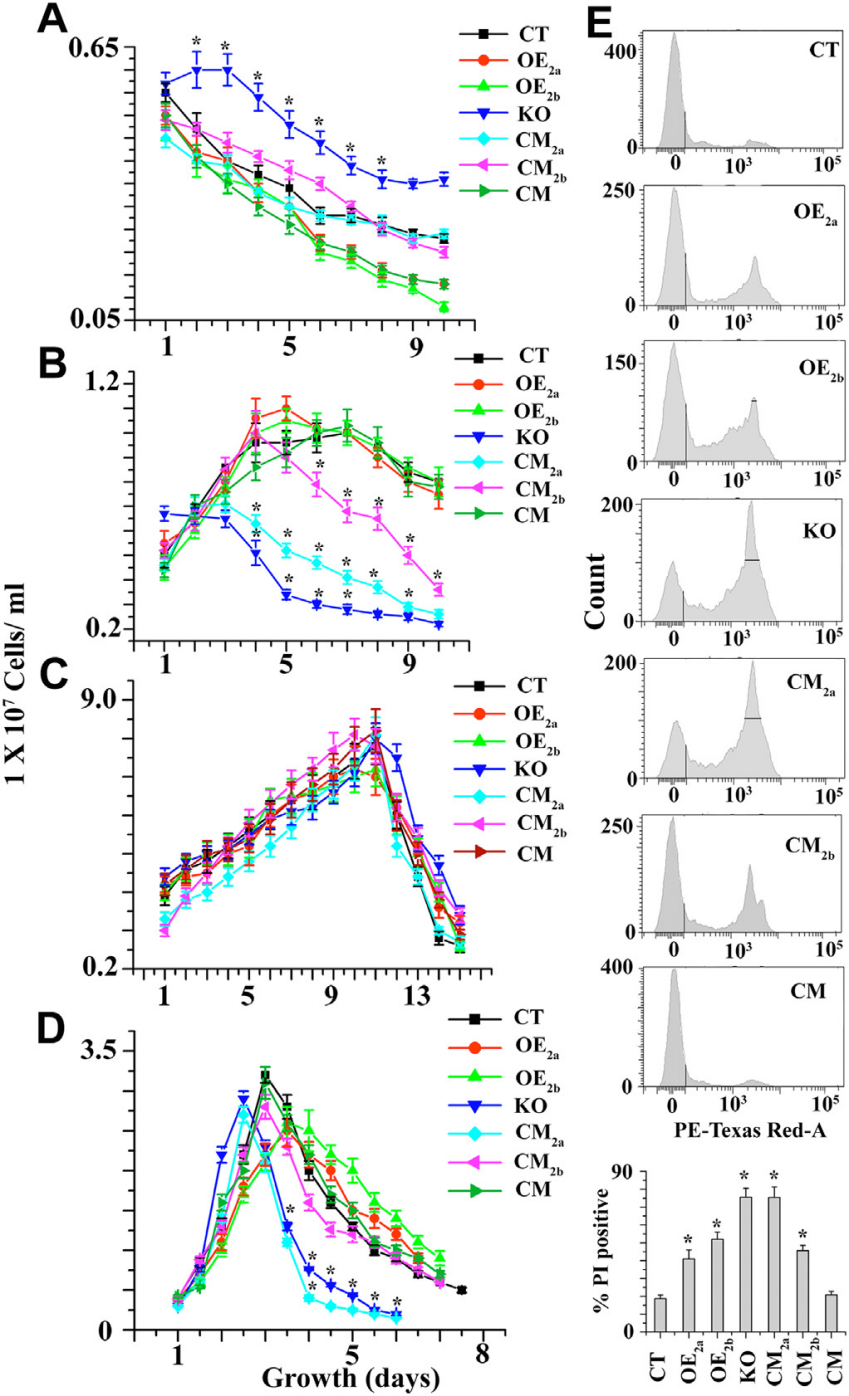
**Comparative studies of the growth profile among CT, OE**_**2a**_**, OE**_**2b**_**, KO, CM**_**2a**_**, CM**_**2b**_**, and CM promastigotes.***A*, 10^6^ mid-log phase promastigotes were seeded in 5 ml sulfur-deficient M199 media (Himedia-AT1076) supplemented with 10% FBS. *B*, 10^6^ mid-log phase promastigotes were seeded in 5 ml sulfur-deficient M199 media supplemented with 10% FBS in presence of 4 mg/ml GSH. *C*, 10^6^ mid-log phase cells were inoculated in 5 ml sulfur-deficient M199 media supplemented with 10% FBS (Gibco) in presence of 25 mg/L N-acetyl cysteine. *D*, 10^6^ mid-log phase promastigotes were seeded in 10 ml normal M199 media supplemented with 10% FBS. *E*, percentage of death cells (PI positive staining) was measured by flow cytometric analysis during the late stationary phase population. ∗Statistically significant value of less than 0.05. FBS, fetal bovine serum; PI, propidium iodide.

The intracellular thiols were increased by providing extracellular GSH in *Leishmania infantum* (31) and GSH supplementation rescued an RNAi knockdown of γ-glutamylcysteine synthetase in *Trypanosoma brucei* (32), indicating that GSH transporter is present in this type of parasite. To investigate whether growth of null mutants in sulfur-limited media is affected by absence or presence of external GSH, growth curve analysis was performed. As expected, the growth curves showed that none of cell lines could grow in sulfur-limited media (Fig. 6*A*). While the KO population could not grow in GSH-containing sulfur-limited media, the OE_2a_, OE_2b_, CT, CM_2a_, CM_2b_, or CM cell line expressing ChaC family proteins grew very efficiently in presence of extracellular GSH (Fig. 6*B*). These results suggest that ChaC family protein in *Leishmania* helps to grow when GSH was used as the only sulfur source. Next, we examined whether null mutants can grow in N-acetyl cysteine (NAC) containing sulfur-limited media. Interestingly, it was observed that null mutants were able to grow in NAC containing sulfur-limited media (Fig. 6*C*). These results indicate that the KO cell can synthesize *de novo* GSH, T(SH)_2_, and other sulfur containing amino acid when NAC was used as the sulfur source. To investigate the role of ChaC2 proteins in the parasite, *Leishmania* were grown in normal nutrient-rich media. KO and CM_2a_ cells had higher growth rates compared to OE_2a_, OE_2b_, CT, CM_2b_, or CM promastigotes (Fig. 6*D*). KO cells were grown faster but the parasite could not survive for longer time in the aged culture media compared to OE_2b_, CT, CM_2b_, or CM cell lines. These results indicate that LmChaC_2b_ protein is required for long-time survival under unfavorable growth condition. According to flow cytometry (Fig. 6*E*) studies, KO cells contained 4-fold (75%) more dead cells (PI-positive cells) than CT (18%) or CM (20%) cells at late stationary phase culture. These results provide an explanation why the growth rate of KO cell was declined rapidly compared to CT cells.

### LmChaC2 is crucial for disease development

**Figure 7:**
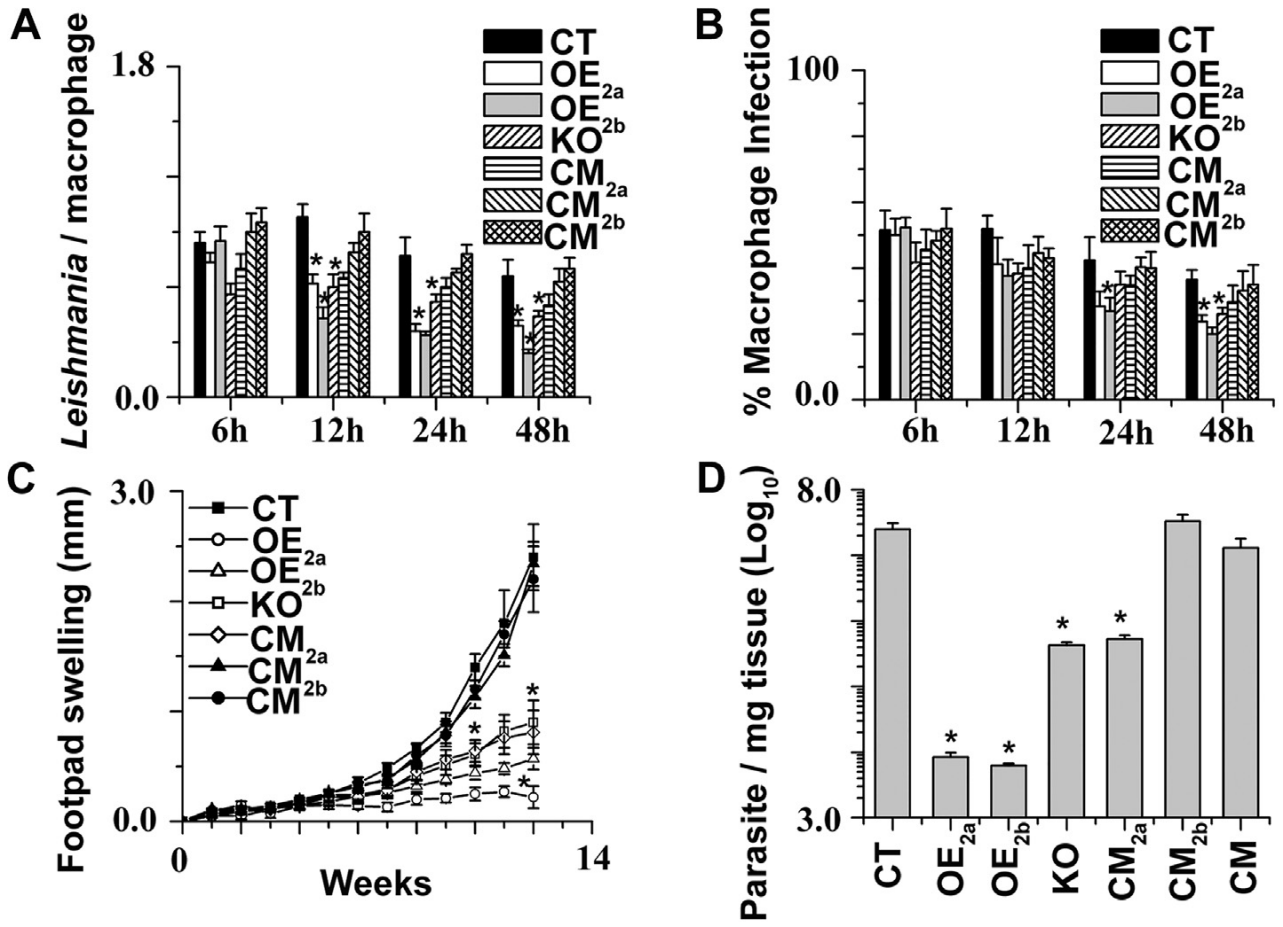
***In vitro* and *in vivo* infection with CT, OE**_**2a**_**, OE**_**2b**_**, KO, CM**_**2a**_**, CM**_**2b**_**, and CM promastigotes.***A*, the number of *Leishmania* within each infected macrophage was counted. For each time point, 200 macrophages were counted. *B*, the percentage of macrophages infected with different LmChaC_2a_ and LmChaC_2b_ variants of parasites. For each time point and cell type, 200 infected macrophages were analyzed. *C*, infection in BALB/c mice. Footpad swelling of different LmChaC_2a_ and LmChaC_2b_ variants was observed for the three groups (8 mice/group). *D*, parasite burden in the footpad after 12 weeks post infection. ∗Statistically significant value of less than 0.05.

*L. major* cells, after infection, grow in host macrophage cells; therefore, we concentrated on the infectivity rate of *Leishmania* promastigotes with the macrophages. We examined to what extent OE_2a_, OE_2b_, CT, KO, CM_2a_, CM_2b_, and CM cells were phagocytosed by the host cells (macrophages). The interaction rates of the KO promastigotes with macrophage were lower compared to those of OE_2a_, OE_2b_, CT, CM_2a_, CM_2b_, or CM cells (Fig. 7*A*). Number of KO parasites in the infected macrophages was still lower compared to control cells when checked after 48 h of incubation. The lower level of infection in KO parasites might be due to lower number of infective metacyclic promastigotes. Additionally, the percentage of macrophages infected with null mutants was also significantly low compared to the CT and CM cell lines (Fig. 7*B*). We discovered that, in contrast to null mutants, CT and CM promastigotes in the BALB/c mice model could develop a severe disease (Fig. 7*C*). On the other hand, the OE_2a_ and OE_2b_ promastigotes were showing very negligible virulence *in vivo* infection model, revealing a fine tuning of the amount of intracellular GSH content is crucial for *Leishmania* infection. To strengthen the aforementioned results, we measured OE_2a_, OE_2b_, CT, KO, CM_2a_, CM_2b_, and CM parasite burden at 12 weeks post infection (Fig. 7*D*). Optimum level of parasite burden was present in CT and CM cells compared to KO and OE cell lines. These data confirmed the importance of LmChaC2 protein–mediated intracellular GSH homeostasis in parasite infection.

## Discussion

It is well known that GSH is essential for *Leishmania* survival, but it is still unknown whether GSH homeostasis in the parasite is required for survival or not. In this article, we have shown for the first time that LmChaC-dependent controlled GSH degradation is crucial for chronic infection. Here, we discuss the following salient points: (a) we demonstrate that both the promastigote and the amastigote stages of *Leishmania* parasites express two ChaC proteins of different molecular weights (LmChaC_2a_ and LmChaC_2b_), which can degrade GSH to produce 5-oxoproline and Cys-Gly dipeptide; (b) the constitutively expressed LmChaC_2a_ (28 kDa) protein has a much lower catalytic efficiency than LmChaC_2b_ (38 kDa); (c) the expression of the LmChaC_2b_ gene was induced under sulfur limitation; (d) the results from null mutants suggest that LmChaC2 proteins control intracellular GSH/T(SH)_2_ concentration, level of oxidative stress, Ca^2+^ concentration, differentiation from noninfective procyclic stage to nondividing infective metacyclic stage and cell death; (e) LmChaC_2b_ gene is required for the long-term parasite survival during stationary phase culture and plays a key role for disease development. Taken together, our data suggest that GSH homeostasis is pivotal for the long-term parasite survival during stationary phase culture and disease development in mice.

Generally, trypanosomatids lack genes like GSH reductase (GR) as well as thioredoxin reductase (TrxR) (33, 34, 35), which are essential for maintaining the intracellular thiol redox homeostasis through the GSH/GR and thioredoxin/TrxR systems in most eukaryotic organisms. On the other hand, trypanosomatids possess a unique pathway of redox metabolism that is based on the low molecular mass dithiol trypanothione [bis(glutathionyl) spermidine], T(SH)_2_ (18) and T(SH)_2_ reductase (36). Therefore, the three main functions of GSH in *Leishmania* parasite are (a) being an essential component in T(SH)_2_ biosynthesis, (b) S-glutathionylation of protein and drugs, and (c) providing cysteine, glycine, and glutamate by degradation. In the GSH cycle of host and parasite, the first two steps for the biosynthesis of GSH from Glu, Cys, and Gly are common in both the organisms. In this article, we showed that the GSH can be degraded by the GSH-specific ChaC-family of γ-GCT (γ-glutamyl cyclotransferase) enzymes to produce cysteinyl-glycine and 5-oxoproline. Like host genes, the *Leishmania* genome sequence have putative 5-oxoprolinase (LmjF.18.1040) and putative M20/M25/M40 family dipeptidase (LmjF.33.1610) genes. The GSH degradation products 5-oxoproline and cysteinyl-glycine are cleaved by 5-oxoprolinase and dipeptidases, respectively, to yield Glu, Cys, and Gly. In contrast to host, parasite’s genome lacks γ-glutamyl transpeptidase (GGT) gene, which has a transpeptidation activity and is involved in the process by which γ-glutamyl group is transferred to the amino acid to produce γ-glutamyl amino acid and cysteinyl-glycine (37). In addition, γ-GCT (C7orf24) and γ-glutamyl amine cyclotransferase (GGACT) are found in mammalian cells; the former enzyme reacts with various γ-glutamyl amino acids to produce 5-oxoproline and amino acids (38) and the latter reacts with γ-glutamyl amine to produce 5-oxoproline and amine (39). However, two parasite specific unique enzymes, glutathionyl-spermidine synthetase and T(SH)_2_ synthetase can consume GSH in presence of spermidine and produce T(SH)_2_, which controls the intracellular redox environment in parasite.

Required for T(SH)_2_ biosynthesis, GSH is an important thiol small molecule for detoxification of ROS and protection against oxidative stress. At par with this, our results show, when GSH degrading enzymes were deleted from the parasite genome, KO cells showed higher intracellular GSH/T(SH)_2_ concentration and subsequently lower level of oxidative stress, free intracellular Ca^2+^ ion and cell death during late log phase. Albeit CT cells had lower growth rate compared to KO promastigotes in nutrient enriched normal media (Fig. 6*D*), CT parasite survive for longer time in the aged culture media compared to KO cell lines, indicating that slower growth was required for long-term survival under unfavorable growth condition. This raises the question as to why slower growth rate was required for long-term survival. One possible explanation is a high rate of replication, as observed in the KO promastigotes, might exploit glycolysis pathway rather than tricarboxylic acid cycle and generate more NADPH and partially oxidized intermediates for the synthesis of excessive amount of DNA, RNA, and proteins and consequently deplete rapidly all essential nutrients from media. Consistent with previous concept, procyclic dividing stage of *Leishmania* promastigotes uses high glucose concentrations, at the same time maintaining controlled flux into the tricarboxylic acid cycle with concomitant production of NADH by secreting partially oxidized intermediates, such as acetate, succinate, and alanine into the culture medium (40). In case of intracellular amastigote stage, a group of researchers had shown from their heavy water labeling experiments that metabolically quiescent state in *Leishmania* parasites in murine lesions may underlie the intrinsic resistance to many stresses (temperature, pH, and nutrient) and is important for slow but constant replication and persistence of the parasite (41, 42). We report here a beneficial role of LmChaC2 proteins in promastigote stage of parasites; LmChaC2-dependent degradation of GSH to Cys, Glu, and Gly may be required for survival in stationary phase culture. Earlier work reports no significant change in the total intracellular thiol concentration during various logarithmic growth phases, but a 6-fold decrease in thiol level is observed as the parasites enter the stationary phase (43). Similarly, the T(SH)_2_ concentration (1–2 mM) remains unaltered during the different logarithmic growth phases of the insect stages of *Trypanosoma cruzi*, *L. major*, and *Leishmania donovani* but decreases rapidly during the stationary phase culture (28). Our results showed that LmChaC_2b_ protein was induced during stationary phase culture and sulfur-limited media (Fig. 3, *C*, *E*, and *F*). Thus, catalytically efficient LmChaC_2b_ protein might be responsible for supplying Cys, Glu, and Gly from GSH that could be utilized for essential protein synthesis during stationary phase, when those nutrients become limited.

T(SH)_2_ and redox proteins of the parasite regulate efficiently the redox homeostasis and withstand the oxidative burst during the early stages of host-*Leishmania* interactions (44). The virulence of LmChaC_2b_ overexpressed cell lines (having negligible amount of T(SH)_2_) is much lower compared to CT cells, indicating that T(SH)_2_ plays an important role in disease development. On the other hand, KO mutants (4-fold excess T(SH)_2_) too have shown lower virulent property than CT. This may suggest that excessive T(SH)_2_ production might as well be harmful to parasite itself. Several other reports have shown deleterious effects of excessive GSH in both mammalian cells and unicellular eukaryotes (8, 9). Increasing GSH concentration by using either addition of NAC or genetic manipulation study (*i.e*., overexpression of glutamate cysteine ligase catalytic subunit (GCLC)/glutamate cysteine ligase modifier subunit (GCLM)) affects the redox status that promotes reductive stress associated with mitochondrial dysfunction (10). Other reports showed that excess GSH is inhibitory to the growth of intracellular human pathogen *Mycobacterium tuberculosis* (45) while its mutant, defective in the uptake of excess GSH, had grown better inside the macrophages (46). They strongly suggested from their observations that this pathogen is exposed to reductive stress within the phagocytic host. In this article, we show that KO mutants defective in the degradation of GSH could not manifest a severe disease, compared to CT promastigotes (Fig. 7*C*). These findings suggest that the generation of cysteine/glutamate/glycine from the recycling of GSH could be vital for protein synthesis and is of significance to the parasite.

## Experimental procedures

### Materials

*Leishmania* strain bank of our Institute provides *L*. *major* (strain 5ASKH) cells. All chemicals were procured from Sigma or sources reported previously (47, 48, 49, 50).

### Ethics statement

Our institutional animal facility provided all BALB/c mice and rabbits and was taking care of all animals. The research project was approved by the IICB Animal Ethical Committee (Registration No.147/1999, CPCSEA). Our institutional animal facility was registered with the Committee for the Purpose of Control and Supervision on Experiments on Animals (CPCSEA), Government of India. The BALB/c mice and rabbits were handled according to their guidelines.

### Cloning of LmChaC_2a_ and LmChaC_2b_

By using QIAamp DNA mini kit, gDNA from promastigotes stage of *L. major* was purified. Primer 1 and primer 2 (Table S1) were used to amplify 714-bp containing LmChaC_2a_ ORF fragment by PCR. The PCR product was digested and cloned using NdeI/BamHI restriction enzymes in the pET28a vector. DNA sequencing was carried out to confirm the ORF. The entire LmChaC_2b_ ORF was similarly amplified by PCR using primer 3 and primer 4 (Table S1) to generate a 972 bp fragment that was digested and cloned into the pET16b vector between NdeI/BamHI restriction sites and confirmed the ORF by DNA sequencing.

### Expression and purification of LmChaC_2a_ and LmChaC_2b_

Recombinant pET28a/LmChaC_2a_ and pET16b/LmChaC_2b_ construct were transformed into *E. coli* BL21 (DE3) and *E. coli* C41 (DE3) cells to express LmChaC_2a_ and LmChaC_2b_ proteins, respectively. Recombinant pET28a/LmChaC_2a_ and pET16b/LmChaC_2b_ transformed cells were inoculated in separate 50 ml LB broth for overnight at 37 °C in the presence of kanamycin (50 μg/ml) and ampicillin (200 μg/ml), respectively. Following overnight growth, cultures were transferred separately into the 500 ml of terrific broth in the presence of respective drug and incubated at 37 °C. About 0.5 mM IPTG was added after attaining the absorbance 0.5 to 0.8 at 600 nm and bacteria were induced for 16 h at 16 °C. Then, the induced cells were harvested by centrifuging at 6000 rpm for 7 min and after washing the pellet with PBS, pellet was resuspended in lysis buffer containing 50 mM potassium phosphate buffer (pH 6.0), 100 mM sodium chloride, glycerol (10%), β-mercaptoethanol, lysozyme (1 mg/ml), imidazole (20 mM), protease inhibitor cocktail without EDTA (Thermo Scientific), and PMSF (1 mM). The complete cell lysis was performed by frequent vortexing, followed by sonication for 20 s pulse and 40 s gap, repeated for 18 times in ice (Ultrasonic homogenizer, model-U250, Takashi). The whole lysate was centrifuged at 14,000 rpm for 1 h and supernatant was loaded into a pre-equilibrated Ni^2+^-NTA column. To obtain the protein from the column, elution buffer was used that contains 250 mM imidazole. Dialysis was performed thrice for removing maximum amount of imidazole from the protein for further analysis. The 10% SDS-PAGE was run to confirm the purity and molecular weight of the proteins.

### Activity measurement by HPLC

Ten micrograms of LmChaC_2a_ or 1.0 μg of LmChaC_2b_ were incubated with 10 mM γ-Glu-Cys, γ-Glu-ε-Lys, GSSG, T(SH)_2_, or GSH in 100 μl of reaction mix containing 50 mM Tris–HCl buffer (pH 8) to examine the γ-GCT activity of the LmChaC proteins. Up to 60 min of incubation at 37 °C, reactions were terminated by heat denaturation at 95 °C for 5 min. Then, denatured proteins were removed by centrifugation at 14,000 rpm for 30 min before injection of the sample in HPLC (Waters). Twenty microliters of the reaction mixture were injected to analyze the reaction products in HPLC using reverse phase C_18_ HPLC column (SunfireTM C_18_ 5 μm 4.6 × 250 mm column, Waters) and 2% perchloric acid as mobile phase (flow rate 1 ml/min, wavelength 210 nm). Authentic standards were used to identify the substrate and all of the products (Cys-Gly, GSH, and 5-oxoproline). Reaction product peaks are appeared at 7.1 min, 8.2 min, and 9.9 min, those are identified as Cys-Gly, oxidized Cys-Gly, and 5-oxoproline, respectively. The concentration of GSH degradation with time was measured by the production of 5-oxoproline from standard curve. Initial velocity of LmChaC proteins was measured at different concentrations of GSH to determine the *K*_*m*_ value for GSH. In case of time-dependent enzymatic reaction, 100 μg of LmChaC_2a_ or 10.0 μg of LmChaC_2b_ were incubated in 1.0 ml of 50 mM Tris–HCl buffer (pH 8) at 37 °C with different concentration of GSH. Hundred microliter aliquots were withdrawn from the reaction mixture at different time intervals. Withdrawal sample was quickly terminated by heat denaturation at 95 °C for 5 min. The enzyme-catalyzed reactions in LmChaC proteins exhibited saturation kinetics. Michaelis–Menten constant (*K*_*m*_) and the turnover number (*k*_cat_) values were calculated by using Michaelis–Menten enzyme kinetics in scientific statistics software (analysis of nonlinear regression) GRAPHPAD PRISM 6, GraphPad Software, Inc.

### Generation of genetically modified T7/Cas9 positive *L. major*

Genetically modified T7/Cas9 positive *L. major* was prepared by transfecting the cells with PacI digested linear pTB007 (Flag::NLS::Cas9::NLSNLS::T7 RNAP) plasmid (51, 52). Cells were serially passaged and maintained in M199 media (Gibco) with 10% fetal bovine serum (FBS) (Gibco) and hygromycin B (100 μg/ml) (Roche). PacI digested linear pTB007 was integrated in the β-tubulin locus of *L. major* for T7 RNA polymerase and Cas9 proteins stable expression. Genetically modified *L*. *major* was identified by PCR method using Cas9 gene specific primer 5 and 6 (Table S1).

### Generation of stable KO strain (LmChaC_2a_ + LmChaC_2b_)^−/−^ for both LmChaC_2a_ and LmChaC_2b_ alleles by CRISPR/Cas9-based editing

CRISPR-Cas9-based editing was performed for KO of both LmChaC_2a_ and LmChaC_2b_ genes in genetically modified *L. major* (strain 5ASKH) promastigote cells (containing T7 polymerase and Cas9). Primer sequences were designed for KO of both LmChaC_2a_ and LmChaC_2b_ (LmjF.22.1190 and LmjF.22.1200) genes by using LeishGEdit server (http://leishgedit.net/). Two repair cassettes bearing the puromycin and the blasticidin-resistance genes were amplified from pTPuro_v1 and pTBlast_v1 vectors, respectively, by using 15 ng pT plasmids, 0.2 mM dNTPs, 3% (v/v) dimethyl sulfoxide, 2 μM of primers 7 and 8 (Table S1), 1 unit platinum Taq DNA polymerase high fidelity (Invitrogen) and 1× high fidelity reaction buffer in 40 μl reaction mixture. PCR steps were initial denaturation at 95 °C for 5 min, then 40 cycles comprise of 30 s at 98 °C, 30 s at 65 °C, and 2 min 15 s at 72 °C, and lastly a final extension of 7 min at 72 °C. Two single-guide RNA (sgRNA) cassettes targeting the 5′ end of LmChaC_2a_ and the 3′ end of LmChaC_2b_ (5′ and 3′ sgRNA templates) were amplified by using 0.2 mM dNTPs, 2 μM of primer 9 (primer G00, sgRNA scaffold), 2 μM of primer 10 (LmChaC_2a_ 5′ sgRNA primers) or primer 11 (LmChaC_2b_ 3′ sgRNA primers) (Table S1), and 1 unit platinum Taq DNA polymerase high fidelity (Invitrogen). PCR steps were initial denaturation for 30 s at 98 °C, followed by 35 cycles of 10 s at 98 °C, 30 s at 60 °C, and 15 s at 72 °C and a final extension step of 10 min at 72 °C. The 1% agarose gel was run to confirm the molecular weight and purity of the PCR products. Two PCR purified drug cassettes, 5′ sgRNA template, and 3′ sgRNA template were cotransfected into log phase T7/Cas9 positive *L. major* cells by electroporation. Electroporation was performed in Bio-Rad Gene Pulsar apparatus using 450 V and 500 μF capacitance. The transfected cells were revived for 24 h in Schneider’s *Drosophila* insect media (Gibco) at 22 °C. After revival at Schneider’s *Drosophila* insect media, cells were transferred in M199 media (Gibco) supplemented with 15% FBS, 100 μg/ml hygromycin B, 20 μg/ml puromycin dihydrochloride (Sigma–Aldrich), and 5 μg/ml blasticidin (Invitrogen) at 22 °C.

### Construction of overexpression system in *L*. *major* (OE_2a_ and OE_2b_)

To amplify LmChaC_2a_ ORF, we used primers 12 and 13 (Table S1). Using primers 14 and 15 (Table S1), entire ORF of LmChaC_2b_ was amplified by PCR. The amplified products were separately inserted into pXG-B2863 vector between SmaI and BamHI sites to construct recombinant pXG-B2863/LmChaC_2a_ and pXG-B2863/LmChaC_2b_. Recombinant pXG-B2863 vector containing LmChaC_2a_ or LmChaC_2b_ was transfected in *L. major* by electroporation using 450 V and 500 μF capacitances with a Bio-Rad Gene Pulsar apparatus. Finally, 200 μg/ml of neomycin was used to maintain all the transfected cells. Western blot analysis was used to confirm overexpressed cell lines with a rabbit anti-LmChaC_2a_ or rabbit anti-LmChaC_2b_ antibody (1:100).

### Complementation of LmChaC_2a_ gene in KO mutant (CM_2a_)

Recombinant pXG-B2863 vector containing LmChaC_2a_ was transfected into the KO cells. Finally, antibiotics neomycin (200 μg/ml), puromycin (20 μg/ml), and blasticidin (5 μg/ml) was used to maintain the transfected CM_2a_ promastigotes at 22 °C. LmChaC_2a_ expression was confirmed by Western blot with rabbit anti-LmChaC_2a_ antibody (1:100).

### Complementation of LmChaC_2b_ in KO mutant (CM_2b_)

The ORF of LmChaC_2b_ was amplified using primer 18 and 19 (Table S1). BamHI site of pXG-PHLEO vector was used to generate recombinant pXG-PHLEO/LmChaC_2b_, which was transfected into the KO cells. Phleomycin (10 μg/ml), puromycin (20 μg/ml), and blasticidin (5 μg/ml) antibiotics was used to maintain transfected cell lines at 22 °C. LmChaC_2b_ expression was measured by Western blot using rabbit anti-LmChaC_2b_ antibody (1:100).

### Complementation of both LmChaC_2a_ and LmChaC_2b_ genes in KO mutant (CM)

LmChaC_2b_/pXG-PHLEO plasmids were transfected into CM_2a_ cells. Antibiotics neomycin, phleomycin, puromycin, and blasticidin (as earlier concentration) was used to maintain the transfected CM cell lines. Expression of both LmChaC_2a_ and LmChaC_2b_ proteins were confirmed by Western blot analysis.

### Production of polyclonal antibodies against LmChaC_2a_ and LmChaC_2b_ proteins

Polyclonal antibodies were generated against the pure recombinant LmChaC_2a_ or LmChaC_2b_ by injecting LmChaC_2a_ or LmChaC_2b_ (20 mg) protein subcutaneously along with Freund's complete adjuvant (Sigma) to a 6-month-old female rabbit, respectively. Next three booster doses of LmChaC_2a_ or LmChaC_2b_ protein (15 mg) along with incomplete adjuvant (Sigma) were administered to respective female rabbit at the interval of 15 days. Two weeks after the last injection, blood was collected from both the ear of rabbits and serum was isolated from the blood by centrifugation at 3000 rpm (20 min) for Western blot analysis.

### Western blot analysis

Protein samples were loaded on 10% SDS-PAGE for separation on the basis of molecular weight and then transferred on 0.45 μm nitrocellulose membrane (Merck-Millipore) by wet transfer for 1 h at 90V with TE series transphor electrophoresis unit (Hoefer) at 4 °C. The 5% bovine serum albumin solution was used to block the membrane for 3 h at 37 °C, after that polyclonal anti-rabbit LmChaC_2a_ or LmChaC_2b_ antibody was applied for overnight incubation at 4 °C. Then membrane was washed thrice with 1× Tris-buffered saline with Tween-20 at 5 min interval and membrane was incubated for 2 h with AP-conjugated anti-rabbit secondary antibody (1:16,000) (Sigma) or horseradish peroxidase (HRP) conjugate secondary antibody (1:16,000) (Merck). Anti-α-tubulin antibody (Merck) and AP or HRP-conjugated antimouse secondary antibody (1:15,000) (Sigma) was used to detect α-tubulin, which was measured as a loading control. Clarity Max TM Western ECL substrate (Bio-rad) and NBT-BCIP (Roche) was applied to develop the band for HRP conjugate antibody and AP conjugated antibody, respectively.

### ROS measurement by CM-H_2_DCFDA

ROS production was measured with CM-H_2_DCFDA (Invitrogen) by flow cytometer. Control, overexpressed, KO, and complement cells (1 × 10^7^ promastigotes/ml) were washed two times with 1× PBS at 4000 rpm (4 °C) for 6 min. Then 6 μM CM-H_2_DCFDA was applied to the cells for 30 min incubation at 26 °C in dark with gentle shaking and fluorescent intensity was measured in the flow cytometer (BD LSR Fortessa) (Ex_λ_-493 nm; Em_λ_-527 nm for CM-H_2_DCFDA).

### Intracellular calcium (Ca^2+^) measurement

Cell permeant calcium indicator Fluo-4/AM (Invitrogen) was used to measure intracellular Ca^2+^ concentrations. About 1 × 10^7^ control, overexpressed, KO, and complement cells were pelleted and washed twice at 4000 rpm for 6 min with chilled 1× PBS. Then, all the cells were incubated separately with 6 μM Fluo-4/AM along with 5 μM pluronic acid F127 for 60 min at room temperature (RT). After that cells were washed twice with fresh media and measured the fluorescence with flow cytometer (BD LSR Fortessa) (Ex_λ_-494 nm; Em_λ_-506 nm).

### Cell death measurement by flow cytometry

DNA intercalating fluorescence dye PI, only permeable to nonliving cells, was used to determine cell death. About 10^7^ control, overexpressed, KO, and complement cells were pelleted down and washed two times with PBS and resuspended in 1× PBS containing PI (5 μg/ml) (Calbiochem) and kept at RT for 15 to 20 min in dark. Fluorescence from the cells was measured by flow cytometer (BD FACS LSR Fortessa) (Ex_λ_-488 nm; Em_λ_-617 nm).

### Growth curve measurement

To investigate the growth profile of different type of cells, 10^6^ mid-log phase control, overexpressed, KO, and complement cells were inoculated in M199 media (10 ml) (Gibco) supplemented with 10% FBS (Gibco). The growth rates of promastigotes were measured by counting cells in Neubauer chamber (hemocytometer) at 24 h interval up to 15 days. To investigate the growth profile of different types of cells in sulfur deficient M199 media, 1 × 10^6^ mid-log phase promastigotes were seeded in 5 ml sulfur-deficient M199 media (Himedia-AT1076) supplemented with 10% FBS in the presence or absence of 4 mg/ml reduced GSH or 25 mg/L NAC (Sigma).

### Intracellular GSH measurement by flow cytometer

Intracellular level of GSH was measured by intracellular GSH Detection Assay Kit (Abcam). About 10^6^ control, overexpressed, KO, and complement cells were taken and washed the cells with twice with 1× PBS at 1500*g* for 6 min. Five microliters of 200× Thiol Green Dye was mixed into 1 ml of cell suspension and incubated at 22 °C for 15 min, then washed the cells at 1000 rpm for 4 min and resuspended in 1 ml of assay buffer. Fluorescence intensity was measured by flow cytometer (BD FACS LSR Fortessa) (Ex_λ_-490 nm; Em_λ_-525 nm).

### Intracellular GSH and T(SH)_2_ measurement by HPLC

About 2 × 10^8^ promastigotes were taken and washed with 1× Dulbecco′s PBS (Sigma) for two times. Then, the cells were resuspended in 200 μl extraction buffer containing 0.1 M HCl and 1 mM EDTA and sonicated for 5 min with vortexing at every 30 s and freeze thaw cycle for two times to ensure cell lysis. Supernatant was collected after centrifuging the samples at 14,000 rpm for 15 min at 4 °C and 40 μl filtered supernatant was injected for HPLC analysis using reverse phase C_18_ HPLC column (Waters) with 2% aqueous solution of perchlorate used as mobile phase (flow rate 1 ml/min, wavelength 210 nm). Peak of GSH and T(SH)_2_ were appeared at 12 min and 52 min, respectively (Fig. S1). GSH and T(SH)_2_ peaks were identified with authentic standards. The intracellular concentration of GSH and T(SH)_2_ was measured from standard curve.

### Macrophage infection

Murine macrophage cell line RAW 264.7 was adhered on the coverslips (2 × 10^6^ macrophages/coverslip) and infected with different LmChaC variant promastigotes cell lines in 0.5 ml of RPMI 1640/10% FBS at 10:1 ratio of parasite to cell in a condition of 5% CO_2_ and 37 °C. Parasite entry was determined after 2 h of infection and intracellular parasite entry was calculated up to 48 h of post infection. After each time-point, unphagocytosed cells were removed by PBS wash and immediately fixed with 100% methanol. Finally, cells were stained with PI and Olympus IX81 microscope was used to visualize and quantify the parasite entry.

### Mice infection

For comparative study of cutaneous infection in mice, 5 × 10^6^ stationary phase promastigotes of different LmChaC variant cells were injected subcutaneously in left hind footpads of 4 to 6 weeks old female BALB/c mice. Daily measurements of footpad swelling with a slide caliper were used to monitor the disease's progression.

### Parasite loads in footpad of mice

Limiting dilution assay was performed to determine the parasite loads in footpad of mice infected with different LmChaC variants. Section of the footpad tissues were homogenized in M199 medium and tissue debris were removed by centrifugation at 500 rpm for 10 min. Then, the supernatant was centrifuged at 4000 rpm for 10 min to pellet down the cells. Pellet was resuspended and serially diluted in M199/10% FBS in a sterile 96-well tissue culture plate. The highest dilution at which the growth of promastigotes could be appeared after 10 to 15 days of incubation at 22 °C was used to calculate the number of visible parasites/mg of tissue.

### Measurement of LmChaC_2a_ and LmChaC_2b_ mRNA expression in promastigotes as well as amastigotes

Semiquantitative reverse transcriptase PCR was performed to measure LmChaC_2a_ and LmChaC_2b_ in promastigote and amastigotes *L. major* cells. RNAqueous-4PCR Kit (Ambion) was used to extract total RNA from different leishmanial cell lines according to manufacturer’s protocol. Complementary DNA (cDNA) was then synthesized from the RNA using Primescript first cDNA synthesis kit (Takara). Primers 1 and 2 was used to amplify LmChaC_2a_, whereas LmChaC_2b_ was amplified using primer 3 and primer 4 to get 714 bp and 972 bp fragment, respectively.

### qRT-PCR for measuring LmChaC_2a_ and LmChaC_2b_ mRNA level in presence of H_2_O_2_, tunicamycin, and sulfur-limited media

Real-time PCR was performed to measure LmChaC_2a_ and LmChaC_2b_ in promastigote after treatment of H_2_O_2_, tunicamycin, and sulfur-limited media. Isolation of total RNA and cDNA synthesis were performed using RNAqueous-4PCR Kit (Ambion) and Primescript first cDNA synthesis kit (Takara), respectively. Faststart Universal SYBR Green Master (Roche) was used to perform real-time quantitative PCR on StepOne Real-Time PCR system (Applied Biosystems) with respective primers. Using 18S rRNA as the endogenous control, the mRNA quantification was performed by the comparative CT method.

### Statistical analysis

The mean ± SD of at least three independent experiments was used to express all results. The Student's *t* test or ANOVA were used to calculate the statistical analysis for the parametric data using Origin 6.0 software (Microcal software, Inc). The difference between individual groups was evaluated using post hoc analysis (multiple comparison *t* test) after the ANOVA. A *p* value of less than 0.05 was accepted statistically significant.

## Data availability

All data generated and analyzed during this study are contained within the article

## Supporting information

This article contains supporting information.

## Conflict of interest

The authors declare that they have no conflicts of interest with the contents of this article.
