# Supplementary material for: The ChaC family of γ-glutamyl cyclotransferases is required for Leishmania to switch to a slow growth state and for long-term survival of the parasite
Source: J Biol Chem. 2022 Sep 17;298(11):102510. doi: 10.1016/j.jbc.2022.102510 (PMC9586994; doi:10.1016/j.jbc.2022.102510)

**Supporting Information for**

**The ChaC family of γ-glutamyl cyclotransferases is required for *Leishmania* to switch to a slow growth state and for long-term survival of the parasite.**

**Sumit Das, Puja Panja, Gaurab Chowdhury, Saroj Biswas, Yuthika Dholey and Subrata Adak***

Division of Structural Biology & Bio-informatics

CSIR-Indian Institute of Chemical Biology

4, Raja S.C. Mullick Road, Kolkata – 700 032

India

* To whom correspondences should be addressed

Tel: +91 33 2473-6793; Fax: +91 33 2473-5197

E-mail: [adaks@iicb.res.in](mailto:alokdatta@iicb.res.in)

**Table S1. Oligonucleotides used in this manuscript.**

| Primer | Primer sequence (underlined indicate restriction sites) | Purpose |
| --- | --- | --- |
| Primer1  Primer2 | 5’- AAAACATATGATGTCATCCTCATCGGAACTG -3’  5’- AAAAGGATCCTCATATCTTCTTTTCGCTACAG -3’ | Cloning of LmChaC_2a_ |
| Primer3  Primer4 | 5’- AAAACATATGATGCCCAAAACTGATACCGAC -3’  5’- AAAAGGATCCTTACGCCATCGTGGCGACC -3’ | Cloning of LmChaC_2b_ |
| Primer5  Primer6 | 5’-AAAAGACAAGAAGTACAGCATCGG-3’  5’-AAAAGTCGCCTCCCAGCTGAGAC-3’ | Conformation of cas9 cassette |
| Primer7  Primer8 | 5’-AAAACAAAGTCGGGAAAAGCAGTAGTAGTCgtataatgcagacctgctgc-3’  5’-GTGGTACAGATATTTTAGCGCACCGTGCCGccaatttgagagacctgtgc-3’ | Repair cassettes |
| Primer9 | 5’-aaaagcaccgactcggtgccactttttcaagttgataacggactagccttattttaacttgctatttctagctctaaaac-3’ | Primer G00 |
| Primer10 | 5’-gaaattaatacgactcactataggTTACATATCTTCGCACATACgttttagagctagaaatagc-3’ | LmChaC_2a_ 5’ sg RNAs primers |
| Primer11 | 5’-gaaattaatacgactcactataggTTTCTCACTTCACCTGTGTGgttttagagctagaaatagc-3’ | LmChaC_2b_ 3’ sg RNAs primers |
| Primer12  Primer13 | 5’-AAAACCCGGGCGCATGTCATCCTCATCGGAACTG-3’  5’-AAAAGGATCCTCATATCTTCTTTTCGCTACAG-3’ | Cloning of OE construct for LmChaC_2a_ |
| Primer14  Primer15 | 5’- AAAACCCGGGCGCATGCCCAAAACTGATACCGAC -3’  5’- AAAAGGATCCTTACGCCATCGTGGCGACC -3’ | Cloning of OE construct for LmChaC_2b_ |
| Primer16  Primer17 | 5’-AAAAGTATAAGCTTGATGGAGAGCG-3’  5’-AAAACAAACTTACGTGTGTGCGTG-3’ | Conformation of knockout strain |
| Primer18  Primer19 | 5’- AAAAGGATCCCGCATGCCCAAAACTGATACCGAC-3’  5’- AAAAGGATCCTTACGCCATCGTGGCGACC -3’ | Cloning of CM Construct for LmChaC_2b_ |

**Supporting Information**

**Figure S1. The velocity of LmChaC2-catalyzed GSH degradation.** 100 µg of LmChaC_2a_ (Panel A) or 10.0 µg of LmChaC_2b_ (Panel B) were incubated in 1.0 ml of 50 mM Tris-HCl buffer (pH-8) at 37°C with different concentration of glutathione. At various time intervals, 100 µl aliquots were taken up from the assay mixture and were terminated by heat denaturation at 95°C for 5 mins. Samples were centrifuged for 30 min to remove the inactivated proteins before HPLC (Waters) analysis. Activity measurement has been described under ‘Experimental Procedures’. All data are fitted to a hyperbolic curve.


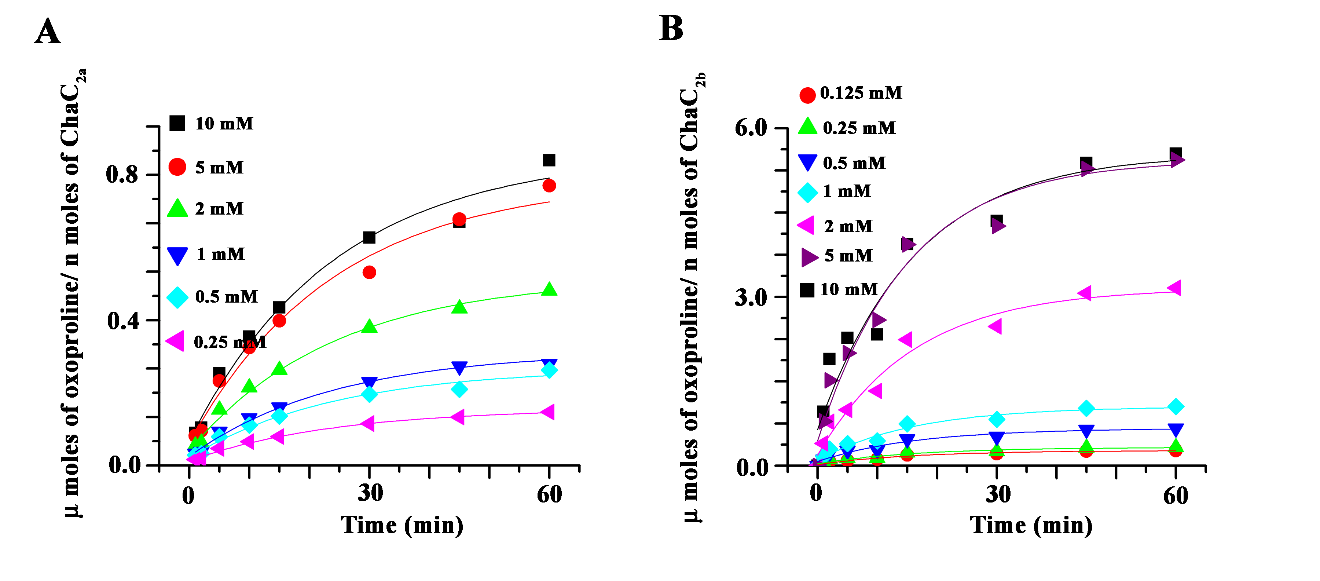


**Supporting Information**

**Figure S2. LmChaC2-catalyzed γ-glutamylcyclotransferase activity for γ-Glu-Cys (Panel A), γ-Glu-ε-Lys (Panel B), GSSG (Panel C) or T(SH)_2_ (Panel D).** Assay system has been described under ‘Experimental Procedures’. The reactions were terminated by heat denaturation at 95°C for 5 min. Samples were centrifuged for 30 min to remove the inactivated proteins before HPLC (Waters) analysis. 20 µl sample was injected to the HPLC system on a SunfireTM C_18_ column (5 µm 4.6 x 250 mm, Waters) and a mobile phase of 2% (v/v) aqueous perchloric acid at 1.0 ml/min.


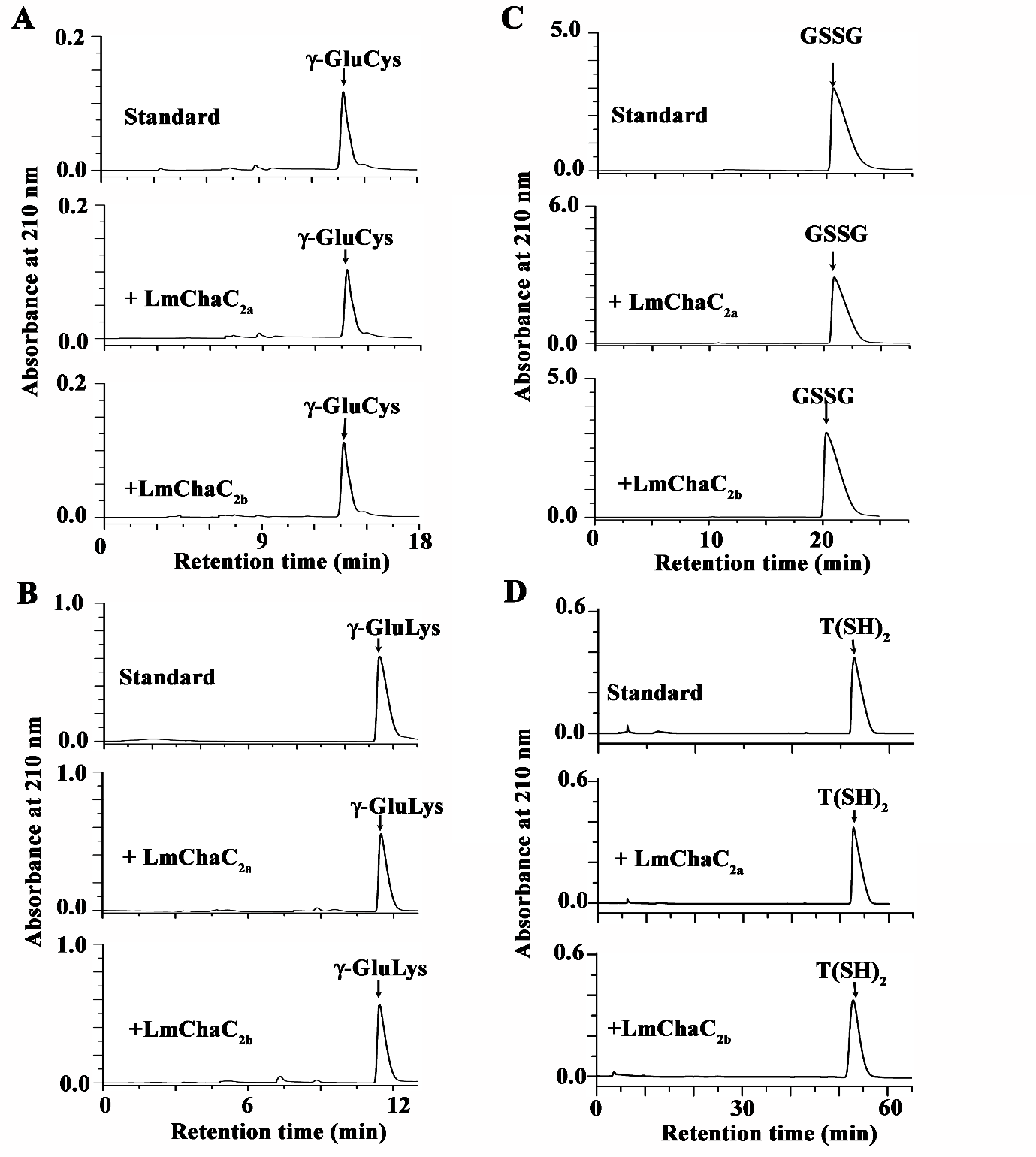


**Supporting Information**

**Figure S3. Intracellular GSH (Panel A) and T(SH)_2_ (Panel B) measurement by HPLC system.** 2x10^8^ promastigotes were taken for making cell lysate. The lysate was centrifuged at 13000g for 10 mins at 4°C and then the supernatant was filtered through 0.22µm Millipore filter paper. 40 µl sample was injected to the HPLC system on a SunfireTM C_18_ column (5 µm 4.6 x 250 mm, Waters) and a mobile phase of 2% (v/v) aqueous perchloric acid at 1.0 ml/min. The concentration of standard Cys-Gly, 5-oxoproline, GSH and T(SH)_2_ are 1.0 mM, 1.0 mM, 1.0 mM and 2.77 mM, respectively.


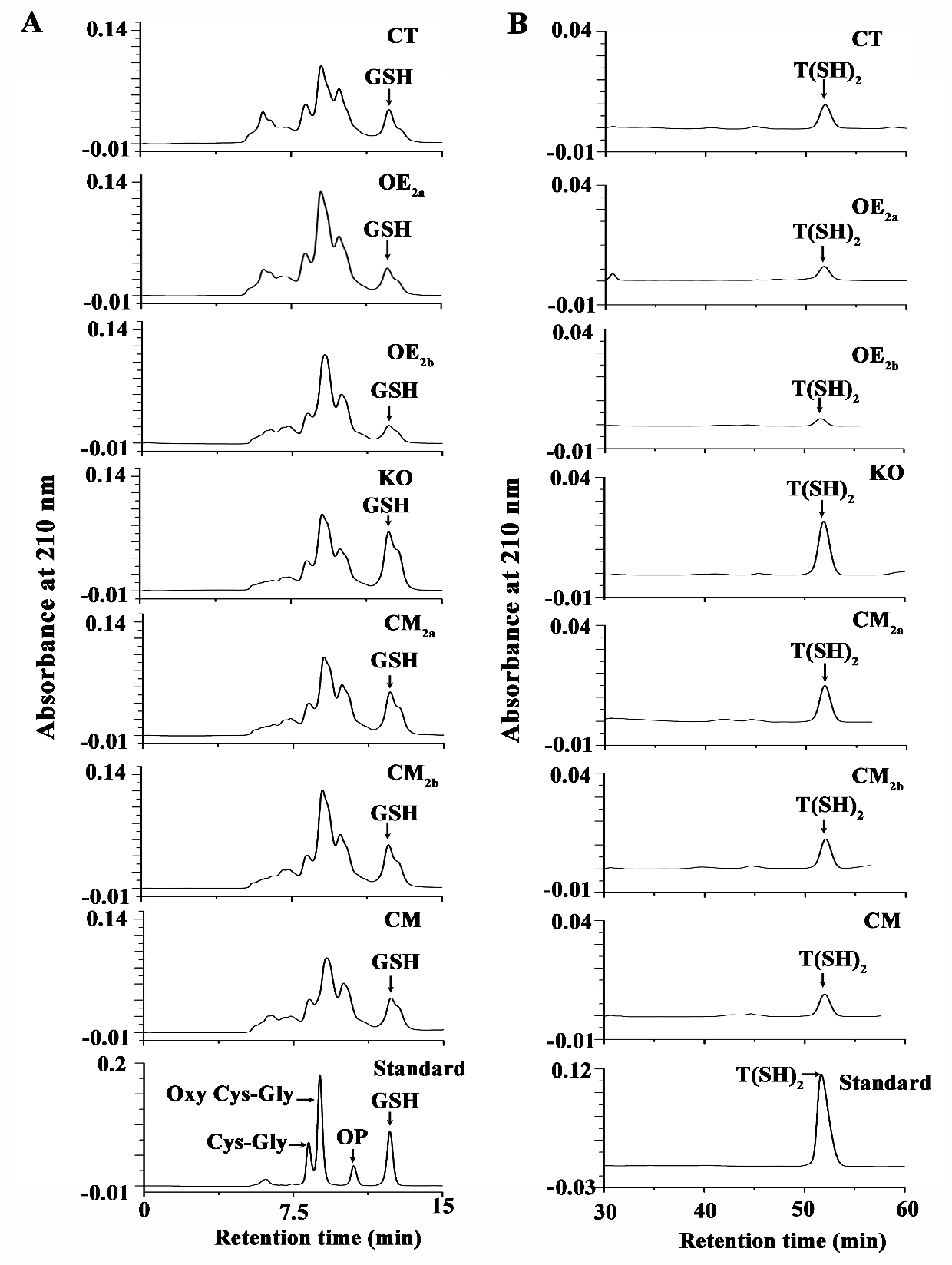

Supplement: Supplemental data [file mmc1.docx]
